# Human Cytomegalovirus Serostatus Defines Cytokine-Based Predictive Signatures in Sepsis

**DOI:** 10.3390/pathogens15020129

**Published:** 2026-01-24

**Authors:** Frederik Krause, Birte Dyck, Kerstin Kappler, Matthias Unterberg, Hartmuth Nowak, Tim Rahmel, Lars Bergmann, Lars Palmowski, Britta Westhus, Alexander Wolf, Alexander von Busch, Barbara Sitek, Patrick Thon, Katharina Rump, Dominik Ziehe, Frank Wappler, Christian Putensen, Stefan Felix Ehrentraut, Alexander Zarbock, Dietrich Henzler, Nina Babel, Martin Eisenacher, Katrin Marcus, Björn Ellger, Björn Koos, Michael Adamzik, Andrea Witowski

**Affiliations:** 1 Ruhr University Bochum, Knappschaft Kliniken University Hospital Bochum, Department of Anesthesiology, Intensive Care Medicine and Pain Therapy, 44892 Bochum, Germany; 2 Ruhr University Bochum, Knappschaft Kliniken University Hospital Bochum, Department of Anesthesiology, Intensive Care Medicine and Pain Therapy, Center for Perioperative Precision Medicine, 44892 Bochum, Germany; 3 Ruhr University Bochum, Knappschaft Kliniken University Hospital Bochum, Department of Anesthesiology, Intensive Care Medicine and Pain Therapy, Center for Artificial Intelligence, Medical Informatics and Data Science, 44892 Bochum, Germany; 4 Ruhr University Bochum, Knappschaft Kliniken University Hospital Bochum, Department of Anesthesiology, Intensive Care Medicine and Pain Therapy, Center for Clinical Proteomic and Metabolomic, 44892 Bochum, Germany; 5 Ruhr University Bochum, Medical Faculty, Medizinisches Proteom-Center, 44801 Bochum, Germany; 6 Department of Anaesthesiology and Critical Care Medicine, Merheim Medical Center, Witten/Herdecke University, 51109 Cologne, Germany; 7 Klinik für Anästhesiologie und Operative Intensivmedizin, Universitätsklinikum Bonn, 53127 Bonn, Germany; 8 Department of Anesthesiology, Intensive Care and Pain Medicine, University Hospital Münster, 48149 Münster, Germany; 9 Klinikum Herford, Department of Anesthesiology, Surgical Intensive Care, Emergency and Pain Medicine, Ruhr University Bochum, 32049 Herford, Germany; 10 Centrum für Translationale Medizin mit Schwerpunkt Immunologie und Transplantation, Marien Hospital Herne, Universitätsklinikum der Ruhr-Universität Bochum, 44625 Herne, Germany; 11 Ruhr University Bochum, Medical Faculty, CUBiMed.RUB, Core Unit Bioinformatics, 44801 Bochum, Germany; 12 Ruhr University Bochum, Medical Proteome Analysis, Center for Proteindiagnostics (PRODI), 44801 Bochum, Germany; 13 Department of Anesthesiology, Intensive Care and Pain Medicine, Klinikum Westfalen, 44309 Dortmund, Germany

**Keywords:** sepsis, cytomegalovirus, cytokine-panels, endotype, latent HCMV infection

## Abstract

(1) Background: Sepsis is characterized by profound heterogeneity of immune responses, complicating biomarker-based prediction of clinical outcomes. Latent human cytomegalovirus (HCMV) infection is one of the strongest modulators of the human immune system and may influence cytokine-mediated signaling during sepsis. (2) Methods: In this post hoc analysis of 331 patients from the prospective multicenter SepsisDataNet.NRW cohort (German Clinical Trial Registry No. DRKS00018871), we quantified 13 serum cytokines on day 1 after sepsis diagnosis and determined HCMV IgG serostatus via ELISA. Using nested cross-validated logistic regression with exhaustive feature selection, we identified cytokine panels predictive of 30-day survival in the total cohort and in subgroups stratified by HCMV serostatus. (3) Results: In the total cohort, a four-cytokine panel (IL-6, IL-10, TNF-α, IL-12p70) predicted 30-day survival with a cross-validated area under the curve (AUC) of 0.66 [95% CI: 0.59–0.72]. Stratification by HCMV serostatus revealed distinct predictive profiles: in HCMV-seropositive patients, a two-cytokine model (IL-10, IL-23) achieved an AUC of 0.69 [95% CI: 0.61–0.77], whereas in seronegative patients, a model based on IL-8 and IL-17A failed to generalize (AUC = 0.47 [95% CI: 0.33–0.61]). Kaplan–Meier analysis confirmed a significant separation of survival curves for the HCMV-seropositive group (*p* < 0.001) but not for seronegative patients (*p* = 0.282). (4) Conclusions: HCMV serostatus defines an immunological context in which cytokine-based prediction of sepsis outcome becomes feasible. These data suggest that viral serostatus should be systematically incorporated into biomarker discovery and immunophenotyping approaches to improve the reproducibility and biological interpretability of sepsis endotyping.

## 1. Introduction

Sepsis is defined as life-threatening organ dysfunction caused by a dysregulated host response to infection [1]. Despite major advances in critical care, sepsis remains one of the most challenging diseases worldwide, with mortality rates of up to 35% [2]. It is among the leading causes of death in industrialized countries, and, to date, no causal therapies exist beyond antibiotic treatment and source control [3].

One major reason for the lack of effective targeted therapies is the high biological heterogeneity of sepsis, which marks the condition as an immunological syndrome rather than a single disease entity. Central to this immunological complexity is the communication between immune cells, largely mediated by cytokines and chemokines.

As key mediators of inflammation, these molecules have long been studied as potential predictors of sepsis onset or outcome, yet with inconsistent results [4,5]. A possible explanation for these divergent findings could be the existence of distinct sepsis subgroups characterized by specific cytokine profiles and variable contributions of individual cytokines [6].

In this context, we recently identified latent human cytomegalovirus (HCMV) infection as a potential modulator of the immune response in sepsis patients [7]. HCMV is an endemic β-herpesvirus with increasing seroprevalence. Like all members of the herpesvirus family, HCMV establishes lifelong latency following primary infection [8]. During latency, the virus may reactivate, causing life-threatening complications in immunocompromised individuals [9,10,11]. Reactivation events, however, also occur intermittently in immunocompetent hosts [12], typically remaining subclinical and thus undetected.

Frequent HCMV reactivation induces a process known as memory inflation, in which CD8^+^ T cells, NK cells, and other immune subsets in seropositive individuals become extensively primed toward viral antigens [13,14]. This long-term reprogramming of the immune system renders latent HCMV infection one of the most powerful immunomodulatory forces known to date [15,16].

Consistent with this, we previously demonstrated that cytokines such as IL-6 and IL-10, well-known prognostic markers in sepsis [4], were predictive of survival only in HCMV-seropositive patients, whereas these associations were absent or markedly weaker in seronegative individuals [7]. These findings led us to hypothesize that cytokine-based prediction models should be stratified by HCMV serostatus to account for virus-induced immune modulation.

We hypothesized that HCMV serostatus defines distinct immunological contexts in sepsis that give rise to different cytokine-driven immune endotypes and thereby modulate the prognostic value of circulating cytokines. To test this hypothesis, we applied an exhaustive logistic-regression approach with cross-validation to identify cytokine panels specifically predictive of 30-day survival in HCMV-seropositive and HCMV-seronegative sepsis patients.

## 2. Materials and Methods

### 2.1. Study Design

This study utilized data from the prospective, multicenter SepsisDataNet.NRW cohort (German Clinical Trials Registry: DRKS00018871), a translational sepsis research network integrating standardized clinical data acquisition, longitudinal biosampling, and interdisciplinary expertise into intensive care medicine, immunology, and bioinformatics across North Rhine–Westphalia, Germany. The overarching aim of SepsisDataNet.NRW is to improve sepsis diagnosis, immunophenotyping, and outcome prediction through data-driven biomarker discovery and systems-level analyses in a real-world clinical setting.

Patients were enrolled between 1 March 2018 and 31 May 2022 from intensive care units (ICUs) at seven university or tertiary care hospitals across North Rhine–Westphalia, Germany. Ethical approval was obtained from the Ethics Committee of the Medical Faculty of Ruhr University Bochum (Registration No. 18-6606-BR) or from the respective local ethics committees at the participating centers.

Eligible patients were adults diagnosed with sepsis within the preceding 36 h, as defined by the sepsis-3 criteria (suspected or confirmed infection plus a SOFA score increase of ≥2 points). Written informed consent was obtained before enrollment. The cohort comprised a heterogeneous population of surgical and medical ICU patients.

Exclusion criteria were (1) age < 18 years at the time of ICU admission, (2) lack or withdrawal of informed consent, and (3) withdrawal of life-sustaining treatment. Patients for whom 30-day survival status could not be determined were excluded from this post hoc analysis.

Biospecimen collection and handling were performed according to standardized operating procedures across all participating centers. Blood samples for cytokine and serological analyses were obtained within a predefined time window of up to 36 h after sepsis diagnosis for sepsis onset measurements. The second and third time points were defined as day 4 and day 8 after sepsis diagnosis. Samples were retrieved immediately after collection, stored under cooled conditions prior to and during transport, and processed using harmonized centrifugation and storage protocols to minimize pre-analytical variability.

### 2.2. Clinical Data and Patient Characteristics

Comprehensive clinical data were extracted from electronic medical records and entered into a centralized research database after pseudonymization in accordance with ethical requirements. Collected variables included vital signs, laboratory parameters, point-of-care diagnostics, demographic data, and ICU length of stay.

To address missing data, experienced physicians at each study site manually reviewed patient charts. When applicable, measurements obtained within ±12 h of sepsis onset were used to complete the dataset.

Severity scores—including the Simplified Acute Physiology Score II (SAPS II) and the Sequential Organ Failure Assessment (SOFA) score—were manually assessed by trained physicians at the respective centers. All patients received treatment according to contemporary sepsis management guidelines.

### 2.3. Measurement of Cytokines

As part of the SepsisDataNet.NRW study, serum samples obtained on day 1 after enrollment were analyzed to quantify 13 cytokines in this study (*n* = 334). For the present post hoc analysis, complete cytokine profiles across all analytes included in the multiplex panel were required to enable exhaustive feature selection and unbiased multivariable modeling. Accordingly, patients with missing values for at least one cytokine (e.g., due to analyte-specific technical failure within the multiplex assay) were excluded. This resulted in the exclusion of 3 additional patients, yielding a final analyzable cohort of *n* = 331 patients.

Cytokine concentrations were measured using the LEGENDplex Human Inflammation Panel 1 (BioLegend, San Diego, CA, USA) following the manufacturer’s instructions. Briefly, serum samples were incubated with LEGENDplex beads for antigen capture, washed, and incubated with detection antibodies. After a final washing step, beads were analyzed on a flow cytometer (Canto II, BD Biosciences, San Jose, CA, USA), and concentrations were calculated from standard curves.

Atypical analyte-specific limits of detection (LODs) are defined by the manufacturer and are provided in the official LEGENDplex Human Inflammation Panel 1 user manual (Bio-Legend). Values below the respective LODs were set to 0 pg/mL and should be interpreted as below-detection measurements rather than absolute concentrations.

### 2.4. Measurement of HCMV Serostatus

HCMV IgG serostatus was determined from day 1 serum samples using the SERION ELISA Classic Cytomegalovirus IgG kit (Institut Virion Serion GmbH, Würzburg, Germany). Diluted samples (1:40, 100 µL) and controls were added to microplate wells and incubated for 60 min at 37 °C. After three washes, 100 µL of substrate solution was added and incubated for at least 20 min at 37 °C. The reaction was stopped by adding 100 µL of stop solution, and optical density (OD) values were recorded using a microplate reader (Sunrise Microplate Reader, Tecan, Männedorf, Switzerland). OD values were normalized to an internal standard, and samples with ≥35 units were considered IgG-positive.

### 2.5. Statistical Analysis

All statistical analyses were performed in Python 3.12 (scikit-learn 1.3, mlxtend 0.22, lifelines 0.27, SciPy 1.11). Medians and 95% confidence intervals (CIs) were estimated by bootstrap resampling (10,000 iterations). Mann–Whitney U tests were adjusted for multiple comparisons using the Benjamini–Hochberg false discovery rate (FDR).

### 2.6. Logistic Regression and Feature Selection

Thirteen serum cytokines measured at enrollment were analyzed (*n* = 331). Depending on the analysis, either the full cohort or subsets stratified by HCMV IgG status were evaluated using identical procedures.

All cytokine variables were standardized using the StandardScaler within each training fold. The scaler was only fit on the respective training data and applied to the corresponding validation fold to avoid data leakage. To identify cytokine signatures predictive of 30-day survival, we implemented a nested cross-validated logistic-regression pipeline with exhaustive feature selection (EFS). Because the models are data-adaptive, inferential *p*-values are not reported; feature stability was quantified by selection frequency.

The outer cross-validation consisted of five stratified folds to obtain out-of-fold (OOF) predictions for all participants. Within each outer training split, an inner five-fold CV performed EFS across cytokine panels of 1 to 5 markers, using the area under the receiver operating characteristic curve (AUC) as the scoring metric. Models were fitted using L2-regularized logistic regression (liblinear, maximum 5000 iterations). All feature selection and hyperparameter tuning were confined to training data within each fold to prevent information leakage.

For each outer fold, a probability cutoff was derived from the inner CV by computing the Youden index for each validation split and taking the median threshold. This fold-specific cutoff was then applied to the corresponding held-out test fold.

### 2.7. Model Performance

For each outer test fold, we calculated AUC and reported fold-level summaries as medians with 95% CIs obtained by percentile bootstrap. In addition, a pooled OOF AUC was computed from all outer-fold test predictions, with 95% CIs estimated by subject-level bootstrap. Kaplan–Meier curves comparing low- and high-survival-score groups were generated from OOF assignments, and survival differences were evaluated using the log-rank test.

### 2.8. Model Interpretability

For each outer fold, we recorded the selected cytokine subset and aggregated selection frequencies across folds. Logistic-regression coefficients were converted to event-oriented odds ratios (ORs) by sign inversion (since the model predicts survival), such that OR > 1 indicates an increased likelihood of death. As coefficients were estimated within multivariable logistic models including multiple cytokines, each OR represents the partial (adjusted) effect of the respective cytokine conditional on the others staying constant. Because all predictors were z-scored, ORs reflect the relative effect per one standard deviation increase. ORs were summarized across folds by median and 95% percentile intervals.

### 2.9. Group-Wise Cytokine Summaries and Testing

Using the OOF-based survival-score assignments, each cytokine was summarized by median and interquartile range (IQR) within the low- and high-survival-score groups. Group differences were tested using two-sided Mann–Whitney U tests, and resulting *p*-values were adjusted for multiple testing by the Benjamini–Hochberg FDR procedure.

All analyses were conducted in Python (scikit-learn, mlxtend, lifelines, SciPy).

## 3. Results

### 3.1. Cohort Description

A total of 331 patients with complete cytokine and 30-day survival data were included in this post hoc analysis. The cohort consisted of 115 female patients (34.74%). The median age of the cohort was 66 years (IQR: 55.5–76). The most common site of infection was the lower respiratory tract (45%), followed by the abdominal cavity (25%). The median SOFA score at inclusion (≤36 h after sepsis diagnosis) was 8 (IQR: 5–11). At sepsis onset, the median serum IL-6 concentration was 212 pg/mL (IQR: 64–575), consistent with published sepsis cohorts (see Supplementary Table S3 for a comprehensive literature comparison [17,18]).

Overall, 232 patients (71.6%) survived the first 30 days, while 99 (28.4%) died. Of all patients, 216 (65.3%) were HCMV-seropositive and 115 (34.7%) were HCMV-seronegative.

When comparing subgroups, HCMV-seropositive patients tended to be older (median 67 years, IQR: 58.25–78 vs. 62.5 years, IQR: 54–73; *p* = 0.009), had comparable disease severity (median SOFA 8, IQR: 6–11.25 vs. 7.5, IQR: 5–10.75; *p* = 0.07), and were more likely to be female (40% vs. 29%; *p* = 0.023).

A detailed overview of baseline characteristics is provided in Table 1.

### 3.2. Cytokine Panels

Exhaustive logistic regression with feature selection of up to five cytokines robustly identified a four-cytokine panel consisting of IL-6, IL-10, TNF-α, and IL-12p70, which predicted 30-day survival in the overall cohort with a cross-validated area under the curve (AUC) of 0.660 [95% CI: 0.594–0.721] based on out-of-fold predictions.

Predicted probabilities of survival were used to categorize patients into two groups: a high-score group, defined by a predicted survival probability below the model-specific cutoff and therefore associated with an increased risk of death, and a low-score group, defined by a survival probability at or above the cutoff and consequently associated with a lower risk of death.

The baseline characteristics for patients categorized in the low or high subgroup are provided in Supplementary Table S4. Patients in the high-score group presented with a higher disease severity at sepsis onset, reflected by a significantly higher SOFA score (median 10 vs. 7, *p* < 0.001), as well as higher levels of procalcitonin and lactate (both *p* < 0.001). In contrast, age, sex distribution, comorbidities, and most infection foci were comparable between groups. Thirty-day mortality was markedly higher in the high-score group (*p* < 0.001).

**Figure 1:**
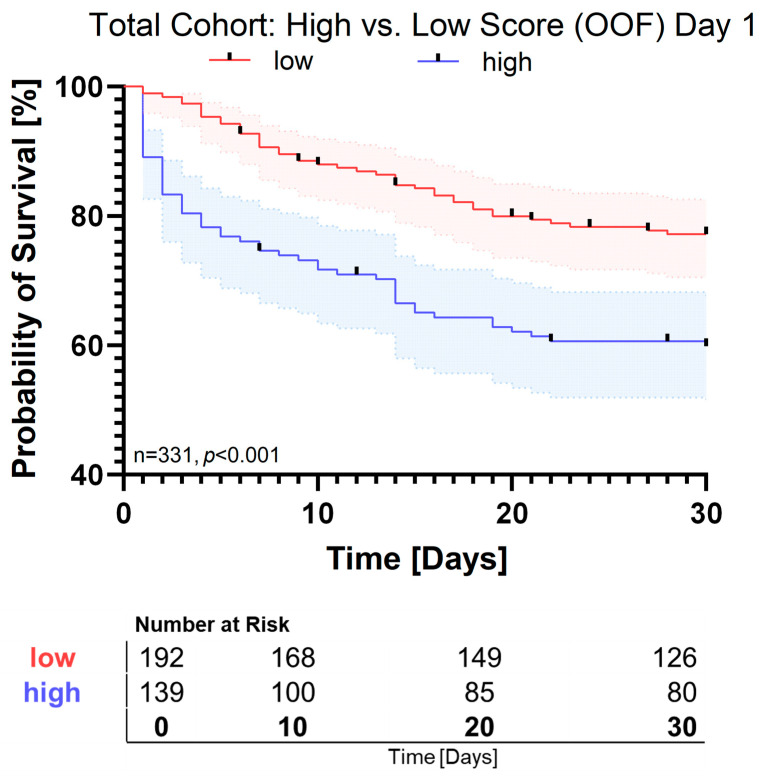
Kaplan–Meier survival analysis of the total cohort based on model-predicted survival scores on day 1. Kaplan–Meier curves illustrate 30-day survival probabilities for patients stratified by model-predicted survival score into low-score (red) and high-score (blue) groups. Survival probabilities are derived from out-of-fold (OOF) predictions obtained in five-fold cross-validation of the logistic-regression model trained on 13 cytokines. Shaded areas indicate 95% confidence intervals. Statistical significance between groups was assessed by log-rank test (*p* < 0.001).

In this model, IL-10 showed the strongest odds ratio (2.49 [95% CI: 2.36–3.29]), followed by IL-6 (1.43 [95% CI: 1.33–1.50]). IL-12p70 was associated with improved survival, showing an odds ratio of 0.46 [95% CI: 0.39–0.82]. Kaplan–Meier analysis demonstrated a clear separation between the high- and low-score groups, with significantly reduced 30-day survival in the high-score group (*p* < 0.001, Figure 1).

Patients in the high-score group showed markedly higher IL-6 concentrations (median 553 pg/mL, IQR: 204–1861) compared with the low-score group (median 108 pg/mL, IQR: 47–280; *p* < 0.001). IL-10 followed a similar pattern, with significantly lower median values in the low-score group (*p* < 0.001).

**Figure 2:**
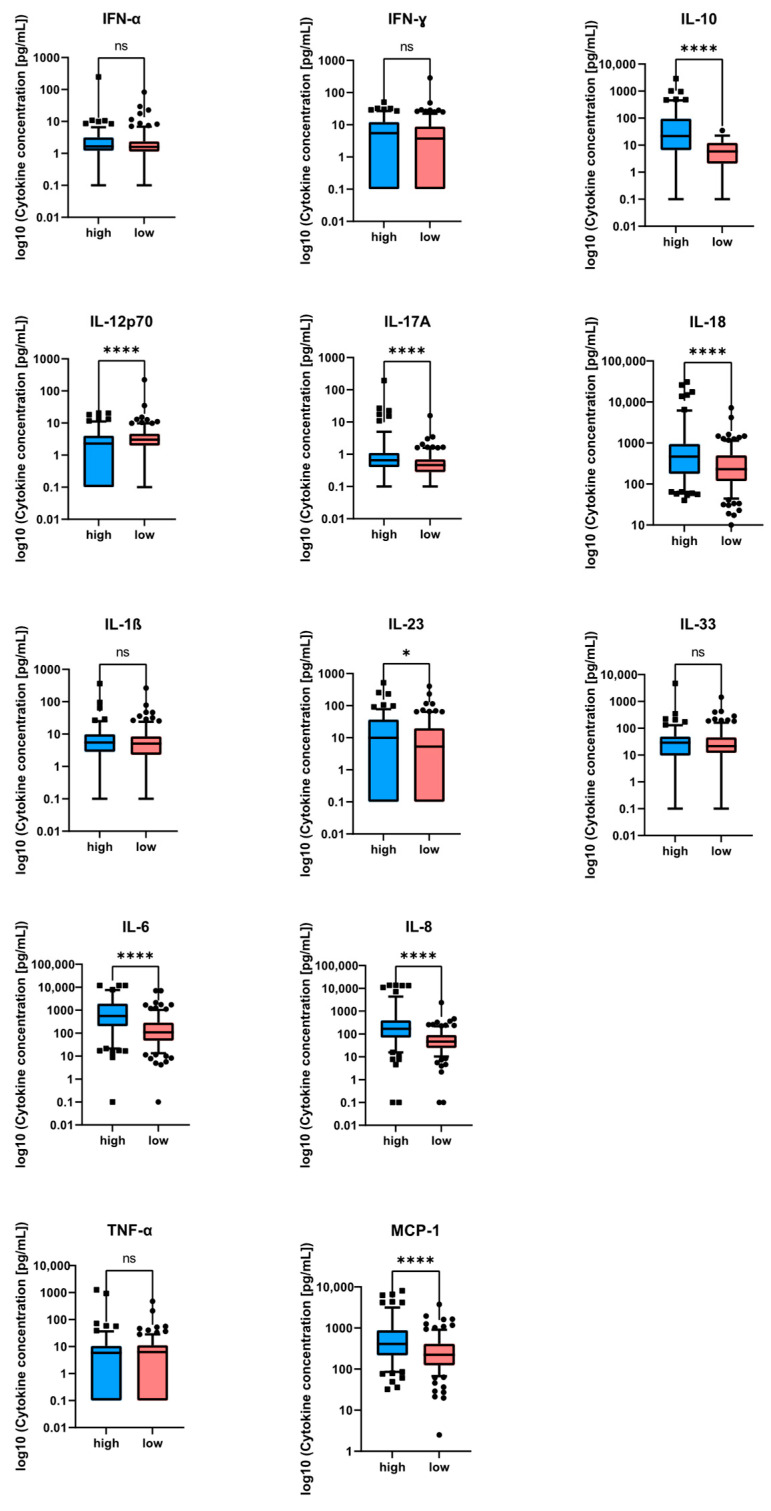
Distribution of 13 serum cytokines in the total cohort at sepsis onset. Boxplots depict log_10_-transformed cytokine concentrations (pg/mL) measured on day 1 after enrollment. Zero values were set to 0.1 prior to log_10_ transformation to enable logarithmic scaling of the *y*-axis. Horizontal lines indicate medians, boxes indicate the interquartile range (IQR), and whiskers indicate the 1.5 × IQR. Statistical comparisons between groups (blue = high-score; *n* = 139 and red = low-score; *n* = 192) were performed using two-sided Mann–Whitney U tests. Resulting *p*-values were adjusted for multiple testing using the Benjamini–Hochberg false discovery rate (FDR) procedure: IL-10, IL-12p70, IL-17A, IL-18, IL-23 IL-6, IL-8, and MCP-1 showed significant differences in the high- and low-score group (ns = not significant; * = *p* < 0.05; **** = *p* < 0.0001).

The complete cytokine distribution for all 13 analytes is summarized in Table 2 and visualized in Figure 2.

### 3.3. HCMV-Seropositive Subgroup

**Figure 3:**
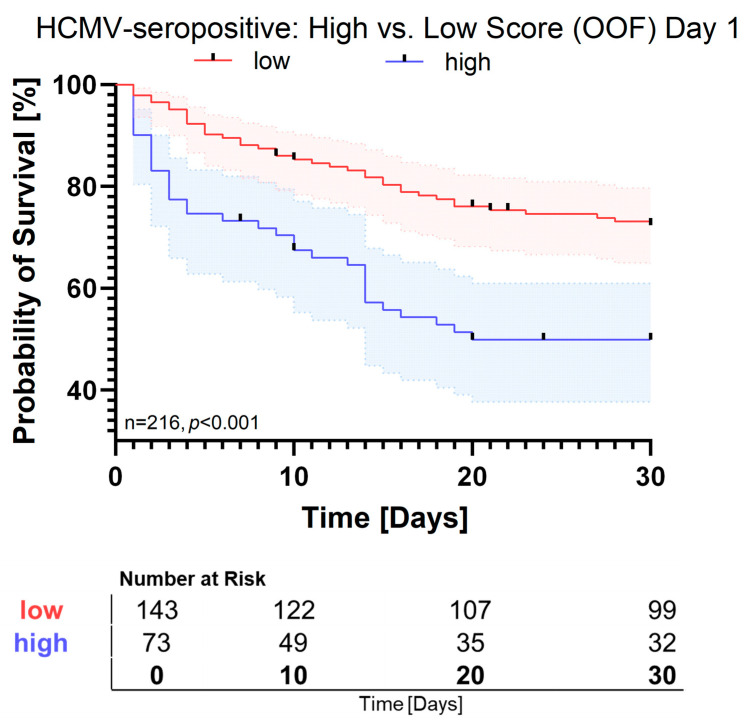
Kaplan–Meier survival analysis of HCMV-seropositive patients based on model-predicted survival scores on day 1. Kaplan–Meier curves depict 30-day survival probabilities for HCMV-seropositive patients, stratified into low-score (red) and high-score (blue) groups according to the logistic-regression model trained on 13 cytokines. Survival probabilities are derived from out-of-fold (OOF) predictions obtained during five-fold cross-validation. Shaded areas represent 95% confidence intervals. A significant difference in survival was observed between groups (log rank *p* < 0.001).

In the HCMV-seropositive subgroup, a two-cytokine panel composed of IL-10 and IL-23 showed robust predictive performance with a cross-validated AUC in the out-of-fold cohort of 0.689 [95% CI: 0.608–0.766]. When applied to cytokines measured at day 4, the algorithm performed similarly well with an AUC of 0.729 [0.628–0.804]. The corresponding Kaplan–Meier curves for day 1 demonstrated a marked survival difference between the high- and low-score groups, which was statistically significant (Figure 3, *p* < 0.001). This result remained stable not only for cytokines at day 1 but also at day 4 (Supplementary Figure S4). IL-10 had a strong impact on survival with an odds ratio of 4.33 [*p* < 0.001, 95% CI: 1.67–7.95] while IL-23 showed a lower odds ratio (1.30 [*p* < 0.001, 95% CI: 1.25–1.48]). IL-10 was again lower in the low-score group (4.6 pg/mL [IQR: 0–9.6]) than in the high-score group (53.0 pg/mL [IQR: 15.5–159.7], *p* < 0.001) (Supplementary Figure S1). IL-23 showed the same pattern with 0 pg/mL [95% CI: 0–13.9] in the low-score group and 20.2 pg/mL [95% CI: 6.1–46.5] (*p* < 0.001) in the high-score group (Supplementary Table S1). Within the HCMV-seropositive subgroup, high-score patients showed significantly higher SOFA scores (median 10.5 vs. 7, *p* < 0.001), higher procalcitonin levels, and elevated lactate concentrations (both *p* < 0.001; Supplementary Table S5). Thirty-day survival was significantly lower in the high-score group (53.1% vs. 74.6%, *p* = 0.002). Demographic variables, comorbidities, and infection foci were largely comparable between groups.

### 3.4. HCMV-Seronegative Subgroup

**Figure 4:**
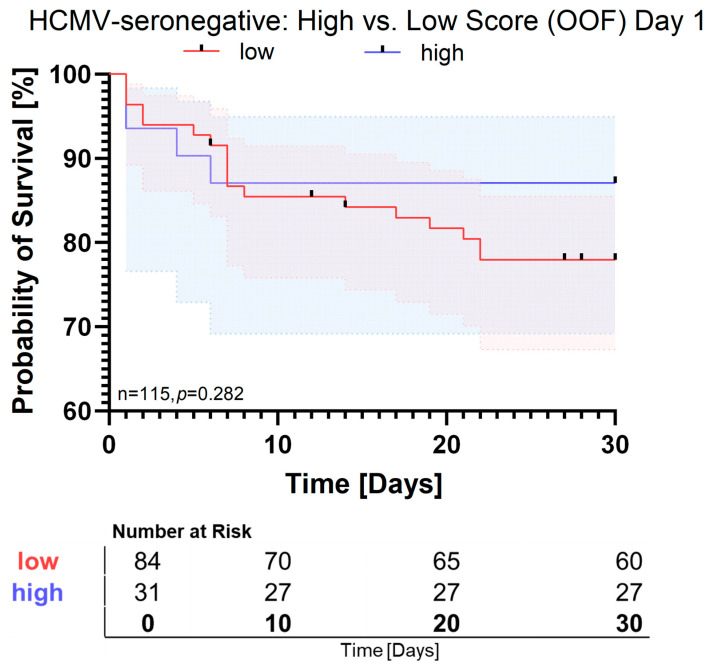
Kaplan–Meier survival analysis of HCMV-seronegative patients based on model-predicted survival scores on day 1. Kaplan–Meier curves depict 30-day survival probabilities for HCMV-seronegative patients, stratified into low-score (red) and high-score (blue) groups according to the logistic-regression model trained on 13 cytokines. Survival probabilities are derived from out-of-fold (OOF) predictions obtained during five-fold cross-validation. Shaded areas represent 95% confidence intervals. No significant difference was observed between groups (log rank *p* = 0.282).

In contrast, analysis of HCMV-seronegative patients yielded a two-cytokine panel of IL-8 and IL-17A with a cross-validated AUC of 0.757 [95% CI: 0.699–0.782] in the training cohort. In the test group, AUC dropped to 0.539 [95% CI: 0.189–0.672]. When applied to the out-of-fold cohort, the AUC dropped further to 0.466 [95% CI: 0.326–0.608], indicating low generalizability. When applied to cytokines measured at day 4, the same pattern emerged with an out-of-fold AUC of 0.430 [0.281–0.587]. Kaplan–Meier curves for day 1 in this subgroup showed only a nonsignificant tendency toward reduced survival in low-score patients (log rank *p* = 0.282), indicating very limited predictive value (Figure 4). Similar results were obtained for day 4 (Supplementary Figure S5). The odds ratio for IL-8 was 2.28 [95% CI: 1.72–2.58] while IL-17A seemed to be protective with an odds ratio of 0.48 [95% CI: 0.41–0.53] in this subgroup. Of these, only IL-17A was significantly different between the low- (0.56 pg/mL [95% CI: 0.39–0.89]) and high-score (0.33 pg/mL [95% CI: 0–0.57]) groups (*p* = 0.002) (Supplementary Figure S2). The other 12 cytokines did not differ between high-score and low-score seronegative patients (Supplementary Table S2). However, despite its higher concentration in the low-score group, the effect of IL-17A did not translate into a generalizable signature. Within the HCMV-seronegative subgroup, baseline characteristics were largely similar between high- and low-score patients (Supplementary Table S6). Although high-score patients showed higher lactate and procalcitonin levels at sepsis onset, no significant differences were observed in SOFA score, ICU length of stay, or 30-day survival.

## 4. Discussion

This study highlights the prognostic heterogeneity of cytokine responses in sepsis and underscores the modulatory role of human cytomegalovirus (HCMV) serostatus. Cytokine responses are highly heterogeneous in sepsis, and their prognostic relevance remains controversial. Latent HCMV infection has been discussed as a potential modulator of the immune response in sepsis patients [7,16,19].

Building on the findings of Unterberg et al. [7], who demonstrated that the prognostic association of IL-6 and IL-10 with survival was confined to HCMV-seropositive individuals, we extended their single-cytokine approach by applying cross-validated multivariate modeling to the same multicentric, prospective SepsisDataNet.NRW cohort. In this post hoc analysis, we explored whether multivariate cytokine panels could identify reproducible predictive signatures, particularly within the HCMV-seronegative subgroup.

Importantly, HCMV serostatus in this study reflects a history of infection and lifelong viral persistence rather than acute viral replication at the time of sepsis. When referring to latent HCMV infection, we are describing the well-established condition of lifelong carriage characterized by intermittent, often subclinical reactivation events over the course of life. However, latent HCMV infection is known to induce long-term immune remodeling, including memory inflation [20] and altered cytokine responsiveness, which may persist independently of acute viral replication. Our findings therefore support the concept that pre-existing immune imprinting by latent HCMV shapes cytokine-based outcome prediction in sepsis.

We found that while IL-6 and IL-10 remained key predictors in the overall population, only the HCMV-seropositive subgroup yielded a reproducible cytokine signature (IL-10 and IL-23) with robust cross-validated performance.

In contrast, models trained in HCMV-seronegative patients failed to generalize across independent test folds, indicating that within the 13 analyzed cytokines, no consistent combination was predictive of outcome. This lack of reproducibility may partly reflect lower cytokine signal-to-noise ratios or greater interindividual heterogeneity in this subgroup, masking potential predictive relationships within the limited sample size. The use of nested cross-validation minimized overfitting bias and increased model robustness compared to earlier single-marker analyses [7]. Nonetheless, logistic-regression models remain sensitive to class imbalance and correlated predictors. The consistent selection of IL-10 across folds, however, underscores the biological robustness of this feature.

Importantly, baseline comparisons support the clinical plausibility of these findings. In the overall cohort and among HCMV-seropositive patients, a high-score classification was consistently associated with more severe acute disease, as indicated by higher SOFA scores, increased lactate levels, and elevated procalcitonin levels, as well as significantly lower 30-day survival rates. By contrast, these associations were weaker in HCMV-seronegative patients, whose baseline characteristics were similar between the high- and low-score groups, and outcome differences were less pronounced. This pattern suggests that cytokine-based scores capture clinically meaningful immune states, particularly in the context of HCMV seropositivity, rather than merely reflecting baseline demographic or comorbidity-related differences.

With a high global seroprevalence, HCMV is endemic to humans and has co-evolved with its host. This coevolution has resulted in sophisticated mechanisms by which the virus alters or evades immune recognition. Although a strong immune response is mounted during primary infection, HCMV is never fully cleared and instead establishes lifelong latency [8].

During this phase, the virus persists in host cells and can periodically reactivate. Beyond its viral proteins, HCMV also employs microRNAs that suppress host cytokine expression, including IL-1, IL-6, and TNF-α. One of the most remarkable immune-evasion strategies is the expression of a viral homolog of IL-10 (cmvIL-10), which mimics the tertiary structure of human IL-10 and binds to the IL-10 receptor [21,22,23].

As IL-10 is a key regulator of immune responses, cmvIL-10 enables HCMV to modulate inflammation, promote viral persistence, and limit immune-mediated clearance [24,25,26]. Hence, HCMV molecularly mimics the properties of the human IL-10, directly manipulating the immune response. This aligns well with the finding that IL-10 is among the cytokines predicting outcome in HCMV-seropositive patients. These known alterations of the immune system by latent HCMV support the plausibility that HCMV-seropositive patients enter sepsis already in an altered immune state, which pushes them towards a more cytokine-driven septic state.

The concomitant inclusion of IL-23 in the HCMV-seropositive signature may reflect activation of the IL-23/IL-17 axis, a cascade implicated in chronic viral immune stimulation and secondary bacterial defense dysregulation. This interplay could contribute to sustained inflammatory signaling in latently infected individuals.

However, our multivariate feature-selection approach identifies cytokine combinations that provide complementary, non-redundant information for outcome prediction. Thus, statistical association does not imply a causal relationship between the included cytokines and biological effects in patients. Therefore, the inclusion of IL-23 in the HCMV-seropositive signature indicates that IL-23 adds predictive information beyond other cytokines, rather than necessarily reflecting a direct mechanistic link to outcome.

In contrast, immunity of HCMV-seronegative patients could be more naïve and therefore less predefined. It could be argued that in HCMV-seronegative patients, other factors (e.g., comorbidities, infection focus, or metabolic resilience) apart from or in combination with cytokines might influence the septic state, which could potentially increase heterogeneity in this cohort. Further work is needed to elucidate these factors in order to increase our understanding of sepsis in HCMV-seronegative patients.

Our findings extend previous work by refining the understanding of how HCMV serostatus shapes the cytokine signaling landscape in sepsis. While Unterberg et al. demonstrated that HCMV serostatus modifies the association of individual cytokines with survival, our multivariate approach reveals that this effect extends to combinatorial cytokine patterns. The observation that only HCMV-seropositive patients exhibit a reproducible cytokine signature suggests that HCMV-induced immune imprinting constrains the cytokine network in a way that renders outcome prediction feasible. Conversely, the absence of a defined pattern in seronegative patients may indicate greater biological variability or the dominance of alternative, non-cytokine determinants of survival. This interpretation fits well within the broader discussion of sepsis endotypes, and multiple inflammatory phenotypes and immune endotypes have been proposed [6,27,28]. Our data suggest that HCMV seropositivity may act as a strong factor driving endotype differentiation in sepsis.

### Limitations

Several limitations need to be considered. Importantly, while HCMV serostatus reflects chronic infection and long-term immune imprinting, blood-based virological assessments cannot exclude localized or transient viral reactivation at the tissue level. Future validation studies should therefore ideally include longitudinal cytokine profiling in combination with direct quantification of viral reactivation markers to better capture dynamic host–virus interactions over the course of sepsis. Furthermore, this was a post hoc analysis of a multicenter prospective study, and although internal cross-validation was applied, as well as strict training- and test-cohort separation, external validation in independent cohorts is required. Future validation should ideally include longitudinal cytokine profiling and direct quantification of viral reactivation markers to better capture dynamic host–virus interactions over the course of sepsis. Although the entire cohort is relatively, large with 331 patients, the HCMV-seronegative group consisted only of 115 patients, limiting its statistical power.

## 5. Conclusions

In summary, our data demonstrate that HCMV serostatus defines an immunological context in which cytokine-based prediction of sepsis outcome becomes feasible. Incorporating viral serostatus into biomarker and endotype analyses may thus improve the reproducibility and biological interpretability of sepsis immunophenotyping.

## Figures and Tables

**Table 1:** Baseline characteristics of the study cohort stratified by HCMV serostatus.

	Total Cohort	CMV-Negative	CMV-Positive	*p*-Value
*n*	331 (100%)	115 (34.74%)	216 (65.26%)	
Female sex, *n* (%)	118 (35.65%)	32 (29.06%)	86 (40.72%)	0.023
Age, years (IQR)	66 (55–76)	62.5 (54–73)	67.00 (58.25–78)	0.010
SOFA at admission (IQR)	8 (5–11)	7.5 (5–10.75)	8 (6–11.25)	0.068
ICU length of stay, days (IQR)	6.81 (2.53–14.45)	7.33 (3.01–14.6)	5.75 (2.18–14.15)	0.219
30-day survival, *n* (%)	237 (71.60%)	94 (81.73%)	143 (66.20%)	0.006

Laboratory values, day 1				
C-reactive protein, mg/dL (IQR)	16.43 (9.56–26.69)	14.88 (8.69–24.41)	18.4 (9.78–28.73)	0.079
Procalcitonin, ng/mL (IQR)	2.54 (0.49–12.15)	1.66 (0.27–11.16)	2.93 (0.59–12.21)	0.283
Lactate, mmol/L (IQR)	1.38 (0.96–2.04)	1.21 (0.93–1.83)	1.53 (1.00–2.44)	0.033
Leukocytes, ×10^3^/µL (IQR)	13.4 (9.43–18.98)	15.1 (9.78–18.8)	13.05 (8.93–19.18)	0.399

Comorbid condition, *n* (%)	272	96	176	
Hypertension	179 (65.81%)	62 (64.58%)	117 (66.48%)	0.856
Cardiovascular disease	108 (39.71%)	33 (34.38%)	75 (42.61%)	0.231
Chronic obstructive pulmonary disease	34 (12.50%)	14 (14.58%)	20 (11.36%)	0.565
Other lung disease	26 (9.56%)	12 (12.50%)	14 (7.95%)	0.316
Chronic kidney disease	52 (19.12%)	14 (14.58%)	38 (21.59%)	0.214
Malignancies	70 (25.74%)	23 (23.96%)	47 (26.70%)	0.726
Diabetes mellitus	78 (28.68%)	21 (21.88%)	57 (32.39%)	0.091
Obesity	78 (28.68%)	26 (27.08%)	52 (29.55%)	0.773
Dialysis	11 (4.04%)	4 (4.17%)	7 (3.98%)	1.000
Organ transplantation	31 (11.40%)	5 (5.21%)	26 (14.77%)	0.030
Nicotine abuse	52 (19.12%)	22 (22.92%)	30 (17.05%)	0.301
Alcohol abuse	22 (8.09%)	9 (9.38%)	13 (7.39%)	0.732
	
Focus of infection, *n* (%)	299	106	193	
Central nervous system	6 (2.00%)	3 (2.83%)	3 (1.55%)	0.670
Lower respiratory tract	135 (45.15%)	51 (48.11%)	84 (43.52%)	0.521
Skin and soft tissue	14 (4.68%)	2 (1.88%)	12 (6.21%)	0.150
Urinary tract	20 (6.69%)	8 (7.55%)	12 (6.21%)	0.843
Cardiovascular system	16 (5.35%)	4 (3.77%)	12 (6.21%)	0.433
Intra-abdominal	75 (25.25%)	27 (25.47%)	48 (24.87%)	1.000
Musculoskeletal	11 (3.67%)	6 (5.66%)	5 (2.59%)	0.304
Other	18 (6.02%)	5 (4.71%)	13 (6.74%)	0.654
COVID-19	4 (1.33%)	0 (0%)	4 (2.07%)	0.301

Baseline demographic, clinical, and laboratory characteristics of all patients included in the analysis (*n* = 331). Data are presented as median and interquartile range (IQR; q25–q75) for continuous variables and as absolute and relative frequencies for categorical variables. HCMV serostatus was determined by ELISA on day 1 after study inclusion. *p*-values were calculated using the two-sided Mann–Whitney U test followed by false discovery rate (FDR) correction for continuous variables. Analysis of binary variables was performed using a Chi-squared test. Abbreviations: ICU = intensive care unit, SOFA = Sequential Organ Failure Assessment.

**Table 2:** Cytokine distribution for all 13 analytes in the high-score (*n* = 139) and low-score (*n* = 192) groups in the total sepsis cohort.

Cytokines	High-Score Group	Low-Score Group	*p*-Value
IFN-ɣ	5.50 (IQR 0.00–12.11)	3.74 (IQR 0.00–8.82)	0.127
IFN-α	1.65 (IQR 1.22–3.08)	1.60 (IQR 1.14–2.36)	0.104
IL-10	21.81 (IQR 6.54–93.94)	4.64 (IQR 0.00–10.79)	<0.001
IL-12p70	2.29 (IQR 0.00–3.99)	3.02 (IQR 2.04–4.63)	<0.001
IL-17A	0.65 (IQR 0.41–1.07)	0.46 (IQR 0.28–0.69)	<0.001
IL-18	466.68 (IQR 179.39–934.77)	230.38 (IQR 118.85–498.50)	<0.001
IL-1ß	5.51 (IQR 2.85–9.82)	5.08 (IQR 2.29–8.44)	0.108
IL-23	10.67 (IQR 0.00–34.51)	5.26 (IQR 0.00–19.75)	0.019
IL-33	29.11 (IQR 9.71–49.36)	21.62 (IQR 12.04–45.51)	0.365
IL-6	553.96 (IQR 204.40–1861.08)	107.83 (IQR 46.90–279.62)	<0.001
IL-8	169.43 (IQR 69.64–389.13)	46.12 (IQR 24.27–88.90)	0.001
MCP-1	408.75 (IQR 217.74–882.92)	222.93 (IQR 124.31–410.15)	<0.001
TNF-α	5.88 (IQR 0.00–10.58)	6.26 (IQR 0.00–11.18)	0.942

Values are shown as median [pg/mL] (IQR q25–q75). *p*-values were calculated using the two-sided Mann–Whitney U test followed by false discovery rate (FDR) correction for multiple comparisons.

## Data Availability

Reproducible code, Excel workbooks containing all tables and OOF assignments, and scalable vector graphics (SVG) plots are available from the corresponding author upon reasonable request.

## Supplementary Materials

The following supporting information can be downloaded at https://www.mdpi.com/article/10.3390/pathogens15020129/s1. Figure S1: Distribution of 13 serum cytokines in HCMV-seropositive patients at sepsis onset; Figure S2: Distribution of 13 serum cytokines in HCMV-seronegative patients at sepsis onset; Figure S3: Kaplan–Meier survival analysis of the total cohort based on model-predicted survival scores on Day 4; Figure S4: Kaplan–Meier survival analysis of HCMV-seropositive patients based on model-predicted survival scores on Day 4; Figure S5: Kaplan–Meier survival analysis of HCMV-seronegative patients based on model-predicted survival scores on Day 4; Table S1: Cytokine distribution for all 13 analytes in high-score and low-score groups in HCMV-seropositive patients; Table S2: Cytokine distribution for all 13 analytes in high-score and low-score groups in HCMV-seronegative patients; Table S3: Comparison of cytokine concentrations in our sepsis cohort versus published sepsis and control values; Table S4: Baseline characteristic for patients with “High” Score and “Low” Score for the total cohort; Table S5: Baseline characteristic for patients with “High” Score and “Low” Score for the seropositive group; Table S6: Baseline characteristic for patients with “High” Score and “Low” Score for the seronegative group.

## Abbreviations

The following abbreviations are used in this manuscript:

AUC	Area Under the Curve
BD	Becton Dickinson (company)
BMFTR	German Federal Ministry of Research, Technology, and Space
CA	California
CI	Confidence Interval
cmvIL-10	Cytomegalovirus-Encoded Interleukin-10
CV	Cross-Validation
de.NBI	German Network for Bioinformatics Infrastructure
DRKS	German Clinical Trials Register
ELISA	Enzyme-Linked Immunosorbent Assay
ERDF	European Regional Development Fund
FDR	False Discovery Rate
HCMV	Human Cytomegalovirus
ICU	Intensive Care Unit
IgG	Immunoglobulin G
IL	Interleukin
IL-6	Interleukin-6
IL-8	Interleukin-8
IL-10	Interleukin-10
IL-12p70	Interleukin-12 p70 subunit
IL-17A	Interleukin-17A
IL-23	Interleukin-23
LOD	Limit of Detection
NRW	North Rhine–Westphalia
OD	Optical Density
OOF	Out-of-Fold
OR	Odds Ratio
pg/mL	Picograms Per Milliliter
SAPS II	Simplified Acute Physiology Score II
SOFA	Sequential Organ Failure Assessment
SVG	Scalable Vector Graphics
TNF-α	Tumor Necrosis Factor Alpha
USA	United States of America
