# Supplementary material for: Human Cytomegalovirus Serostatus Defines Cytokine-Based Predictive Signatures in Sepsis
Source: Pathogens. 2026 Jan 24;15(2):129. doi: 10.3390/pathogens15020129 (PMC12942782; doi:10.3390/pathogens15020129)
Supplement: Supplementary file 1 [file pathogens-15-00129-s001.zip › pathogens-4071101-supplementary.pdf]

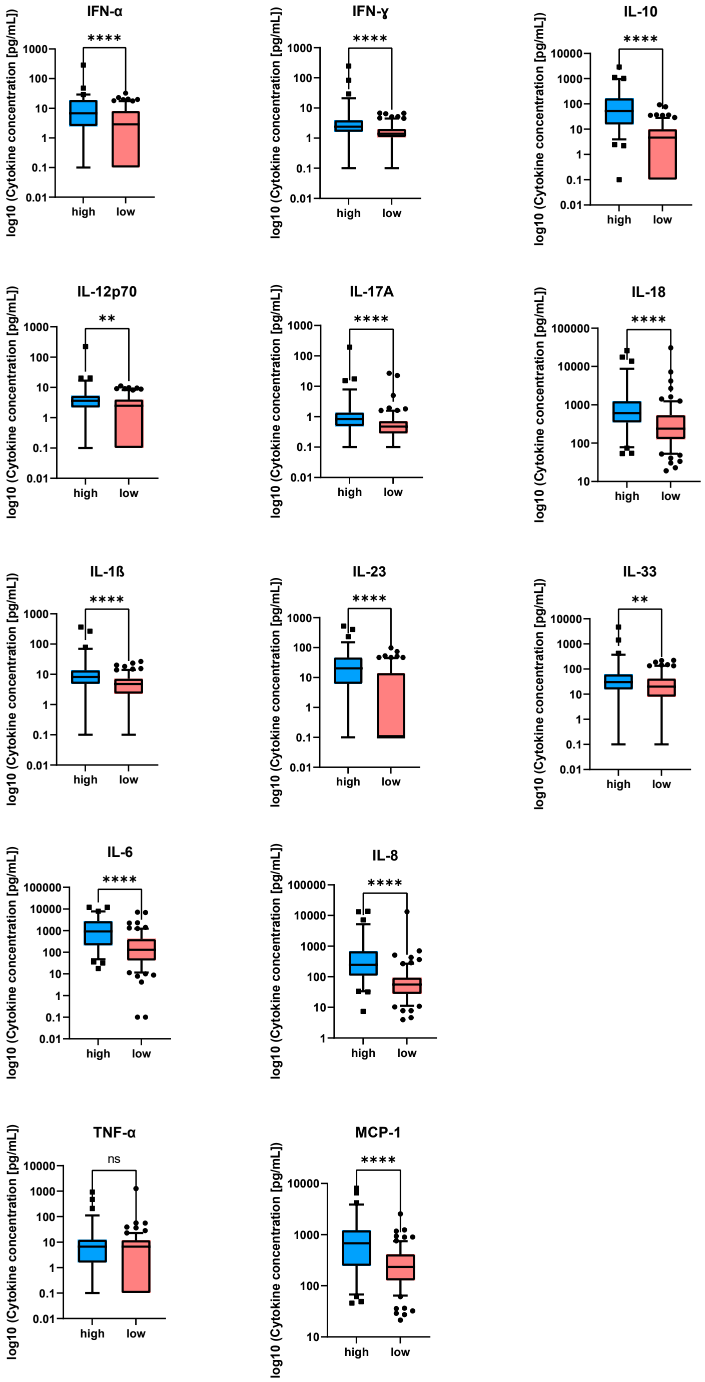

Supplementary Figure S1: Distribution of 13 serum cytokines in HCMV-seropositive patients at sepsis onset. Boxplots depict log_{10}-transformed cytokine concentrations (pg/mL) measured on day 1 after enrollment. Zero values were set to 0.1 prior to log_{10} transformation to enable logarithmic scaling of the y-axis. Horizontal lines indicate medians, boxes the interquartile range (IQR), and whiskers the 1.5 × IQR range. Statistical comparisons between groups (blue = high-score n=73 and red = low-score n=143) were performed using two-sided Mann–Whitney-U-tests: TNF-α, IFN-ɣ, IL-10, IL-12p70, IL-17A, IL-18, IL-1ß, IL-23, IL-33, IL-6, IL-8 and MCP-1 showed significant differences in high- and low-score group (ns = not significant; * = $p < 0.05$; ** = $p < 0.005$; **** = $p < 0.0001$).

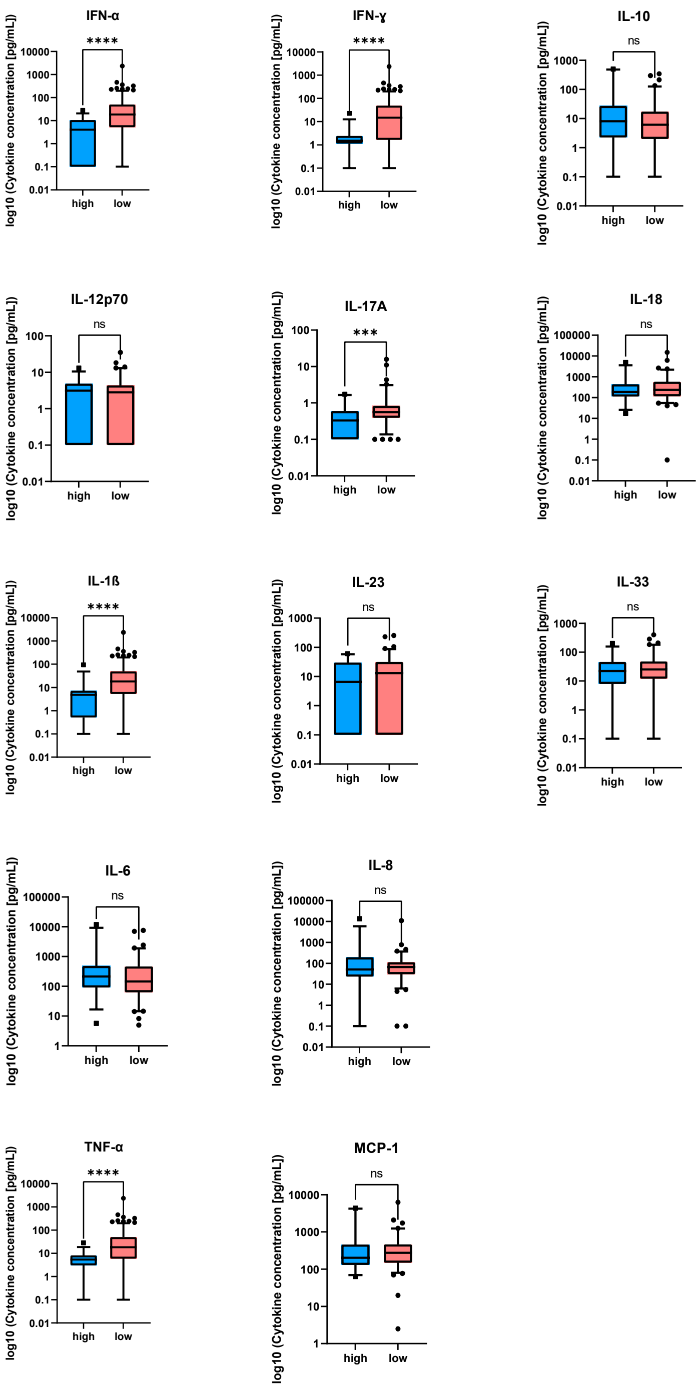

Supplementary Figure S2: Distribution of 13 serum cytokines in HCMV-seronegative patients at sepsis onset. Boxplots depict $\log_{10}$-transformed cytokine concentrations (pg/mL) measured on day 1 after enrollment. Zero values were set to 0.1 prior to $\log_{10}$ transformation to enable logarithmic scaling of the y-axis. Horizontal lines indicate medians, boxes the interquartile range (IQR), and whiskers the 1.5 × IQR range. Statistical comparisons between groups (blue = high-score n=31 and red = low-score n=84) were performed using two-sided Mann–Whitney-U-tests: IL-17A showed significant differences in high- and low-score group (ns = not significant; *** = $p < 0.0005$)

Supplementary Table S1: Cytokine distribution for all 13 analytes in high-score (*n*=73) and low-score (*n*=143) groups in HCMV seropositive patients at sepsis onset. Values are shown as median [pg/ml] (IQR q25–q75). P-values were calculated using the two-sided Mann–Whitney U test followed by false discovery rate (FDR) correction for multiple comparisons.

Cytokines	High-score	Low-score	p-value
IFN-ɣ	2.39 (IQR 1.64–3.93)	1.40 (IQR 1.07–1.98)	<0.001
IFN-α	6.79 (IQR 2.53–18.16)	2.90 (IQR 0.00–8.03)	<0.001
IL-10	53.03 (IQR 15.52–159.73)	4.64 (IQR 0.00–9.63)	<0.001
IL-12p70	3.61 (IQR 2.29–5.27)	2.47 (IQR 0.00–3.99)	0.002
IL-17A	0.84 (IQR 0.50–1.34)	0.47 (IQR 0.29–0.71)	<0.001
IL-18	605.68 (IQR 359.65–1222.85)	239.40 (IQR 127.59–536.66)	<0.001
IL-1ß	8.14 (IQR 4.85–13.26)	4.79 (IQR 2.29–7.18)	<0.001
IL-23	20.18 (IQR 6.13–46.47)	0.00 (IQR 0.00–13.86)	<0.001
IL-33	30.08 (IQR 15.86–58.90)	20.09 (IQR 7.91–40.40)	0.004
IL-6	908.78 (IQR 215.65–2537.77)	130.20 (IQR 41.14–405.14)	<0.001
IL-8	246.05 (IQR 110.18–675.81)	56.11 (IQR 27.86–93.77)	<0.001
MCP-1	676.11 (IQR 247.87–1188.12)	233.75 (IQR 129.03–412.60)	<0.001
TNF-α	6.62 (IQR 3.05–12.02)	6.62 (IQR 0.00–11.66)	0.62

Supplementary Table S2: Cytokine distribution for all 13 analytes in high-score (*n*=31) and low-score (n=84) groups in HCMV seronegative patients at sepsis onset. Values are shown as median [pg/ml] (IQR q25–q75). P-values were calculated using the two-sided Mann–Whitney U test followed by false discovery rate (FDR) correction for multiple comparisons.

Cytokines	High-score	Low-score	p-value
IFN-ɣ	1.50 (IQR 1.20–2.28)	1.60 (IQR 1.16–2.27)	0.43
IFN-α	4.08 (IQR 0.00–9.73)	4.88 (IQR 2.31–9.20)	0.73
IL-10	8.16 (IQR 2.27–21.75)	6.13 (IQR 2.01–17.29)	0.50
IL-12p70	3.13 (IQR 0.92–4.83)	2.82 (IQR 0.00–4.29)	0.57
IL-17A	0.33 (IQR 0.00–0.57)	0.56 (IQR 0.39–0.82)	<0.001
IL-18	187.29 (IQR 115.91–410.44)	235.24 (IQR 116.19–559.87)	0.44
IL-1ß	4.79 (IQR 0.76–6.99)	5.13 (IQR 2.82–9.62)	0.27
IL-23	6.52 (IQR 0.00–26.61)	13.12 (IQR 0.00–30.85)	0.31
IL-33	22.19 (IQR 8.79–45.59)	25.60 (IQR 12.04–47.48)	0.68
IL-6	211.60 (IQR 98.98–473.17)	145.29 (IQR 63.76–447.79)	0.33
IL-8	50.78 (IQR 23.76–190.28)	66.69 (IQR 30.57–108.99)	0.63
MCP-1	202.83 (IQR 130.52–441.25)	276.31 (IQR 150.29–466.09)	0.39
TNF-α	5.43 (IQR 3.12–7.68)	4.95 (IQR 0.00–10.49)	1.00

Supplementary Table S3: Comparison of cytokine concentrations in our sepsis cohort versus published sepsis and control values. Our cohort values are shown for HCMV-seropositive patients at sepsis onset (day 1), stratified into high-score (*n*=139) and low-score groups (*n*=192) (median and IQR). Published comparator values are provided as reported in the original studies [17,18]. Units are pg/mL. Note that absolute concentrations may differ across studies due to assay platforms, calibration standards, sample type, and timing of blood draws.

Cytokine (pg/mL)	Our cohort HCMV+ high-score (median, IQR)	Our cohort HCMV+ low-score (median, IQR)	Literature sepsis (mean, SD)	Literature controls (mean, SD)	Reference
**IFN-γ**	5.50 (IQR 0.00–12.11)	3.74 (IQR 0.00–8.82)	20.82 ± 142.23	0.00 ± 0.00	Jekarl et al., 2019
**IFN-α**	1.65 (IQR 1.22–3.08)	1.60 (IQR 1.14–2.36)	7.82 ± 63.18	0.05 ± 0.48	Jekarl et al., 2019
**IL-10**	21.81 (IQR 6.54–93.94)	4.64 (IQR 0.00–10.79)	88.55 ± 321.44	0.07 ± 0.44	Jekarl et al., 2019
**IL-12p70**	2.29 (IQR 0.00–3.99)	3.02 (IQR 2.04–4.63)	0.36 ± 2.39	0.19 ± 1.72	Jekarl et al., 2019
**IL-17A**	0.65 (IQR 0.41–1.07)	0.46 (IQR 0.28–0.69)	7.05 ± 16.59	1.04 ± 2.44	Jekarl et al., 2019
**IL-18**	466.68 (IQR 179.39–934.77)	230.38 (IQR 118.85–498.50)	53.49 ± 85.48	1.50 ± 5.51	Jekarl et al., 2019
**IL-1β**	5.51 (IQR 2.85–9.82)	5.08 (IQR 2.29–8.44)	3.26 ± 7.92	0.45 ± 1.62	Jekarl et al., 2019
**IL-23**	10.67 (IQR 0.00–34.51)	5.26 (IQR 0.00–19.75)	58.33 ± 272.71	2.11 ± 15.13	Jekarl et al., 2019
**IL-33**	29.11 (IQR 9.71–49.36)	21.62 (IQR 12.04–45.51)	40.3 (sepsis/septic shock) vs 27.5 (nonseptic)	not reported as healthy controls in this study	Chun et al., 2018
**IL-6**	553.96 (IQR 204.40–1861.08)	107.83 (IQR 46.90–279.62)	1931.93 ± 4803.73	4.72 ± 17.12	Jekarl et al., 2019
**IL-8**	169.43 (IQR 69.64–389.13)	46.12 (IQR 24.27–88.90)	310.08 ± 945.84	12.43 ± 36.10	Jekarl et al., 2019
**MCP-1**	408.75 (IQR 217.74–882.92)	222.93 (IQR 124.31–410.15)	811.06 ± 1335.39	65.96 ± 48.52	Jekarl et al., 2019
**TNF-α**	5.88 (IQR 0.00–10.58)	6.26 (IQR 0.00–11.18)	4.43 ± 14.96	1.64 ± 5.73	Jekarl et al., 2019

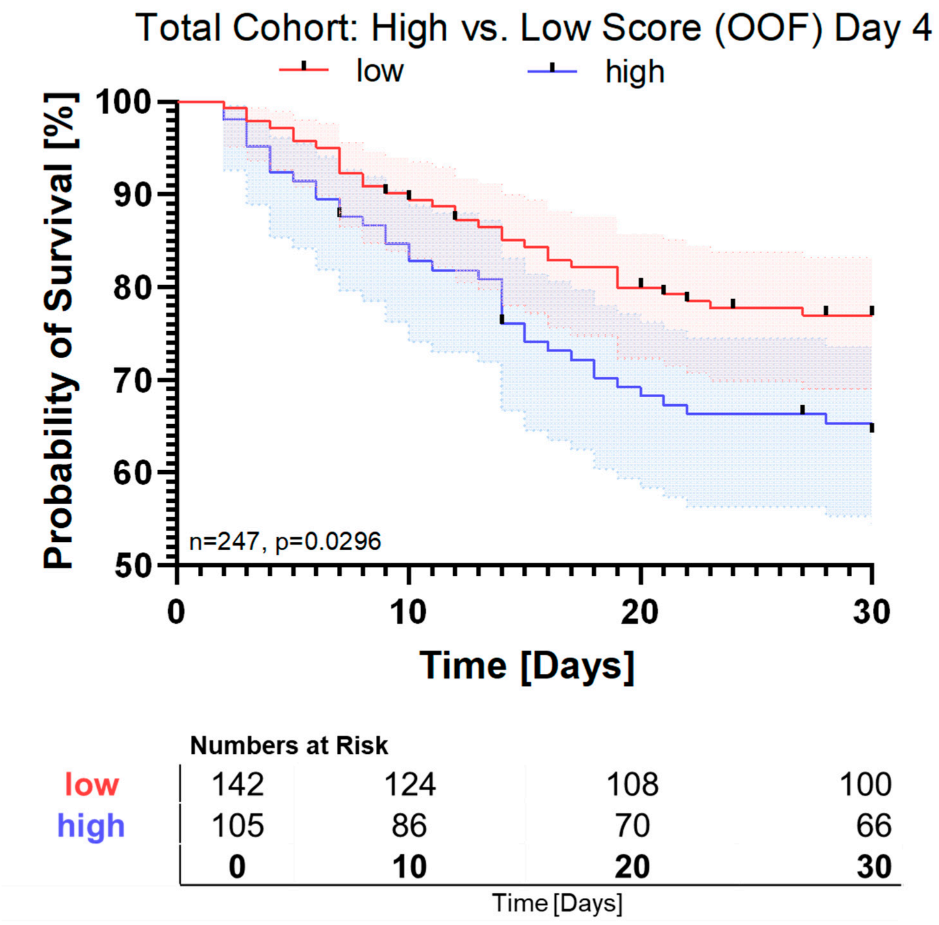

Supplementary Figure S3: Kaplan–Meier survival analysis of the total cohort based on model-predicted survival scores on Day 4. Kaplan–Meier curves illustrate 30-day survival probabilities for patients stratified by model-predicted survival score into a low-score (red) and high-score (blue) group. Survival probabilities are derived from out-of-fold (OOF) predictions obtained in five-fold cross-validation of the logistic-regression model trained on 13 cytokines. Shaded areas indicate 95% confidence intervals. Statistical significance between groups was assessed by log-rank test (p = 0.0296).

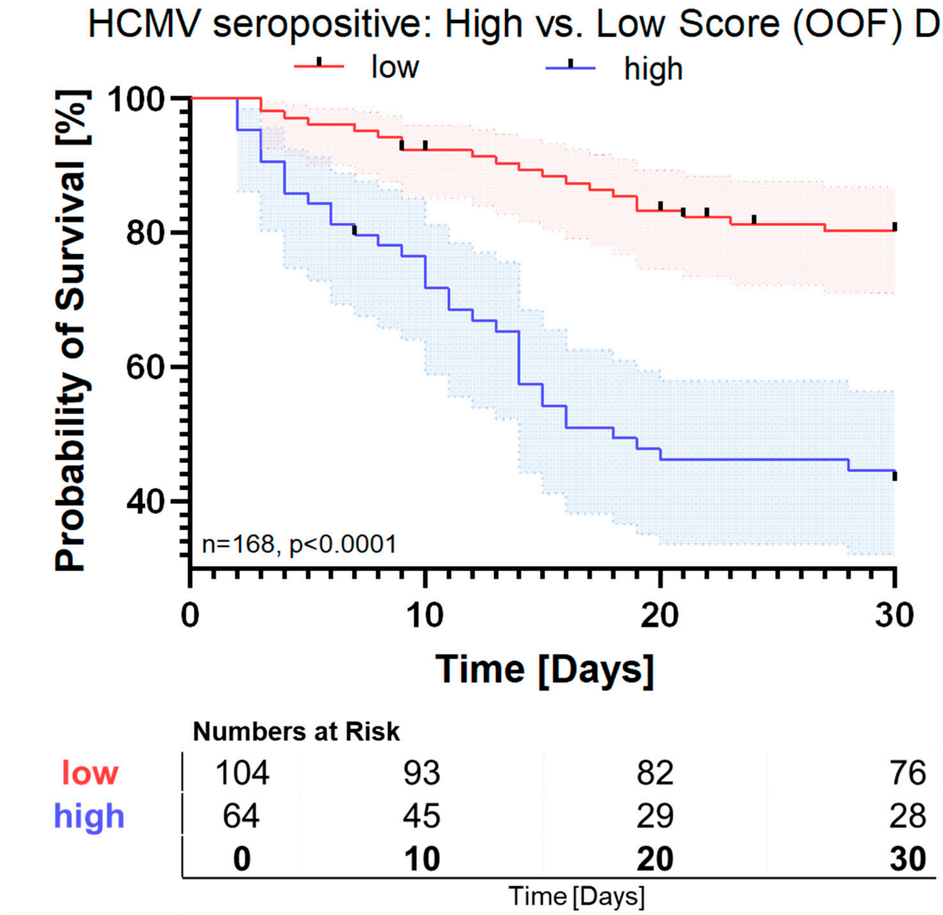

Supplementary Figure S4: Kaplan–Meier survival analysis of HCMV-seropositive patients based on model-predicted survival scores on Day 4. Kaplan–Meier curves depict 30-day survival probabilities for HCMV-seropositive patients, stratified into a low-score (red) and high-score (blue) group according to the logistic-regression model trained on 13 cytokines. Survival probabilities are derived from out-of-fold (OOF) predictions obtained during five-fold cross-validation. Shaded areas represent 95% confidence intervals. A significant difference in survival was observed between groups (log-rank $p < 0.0001$).

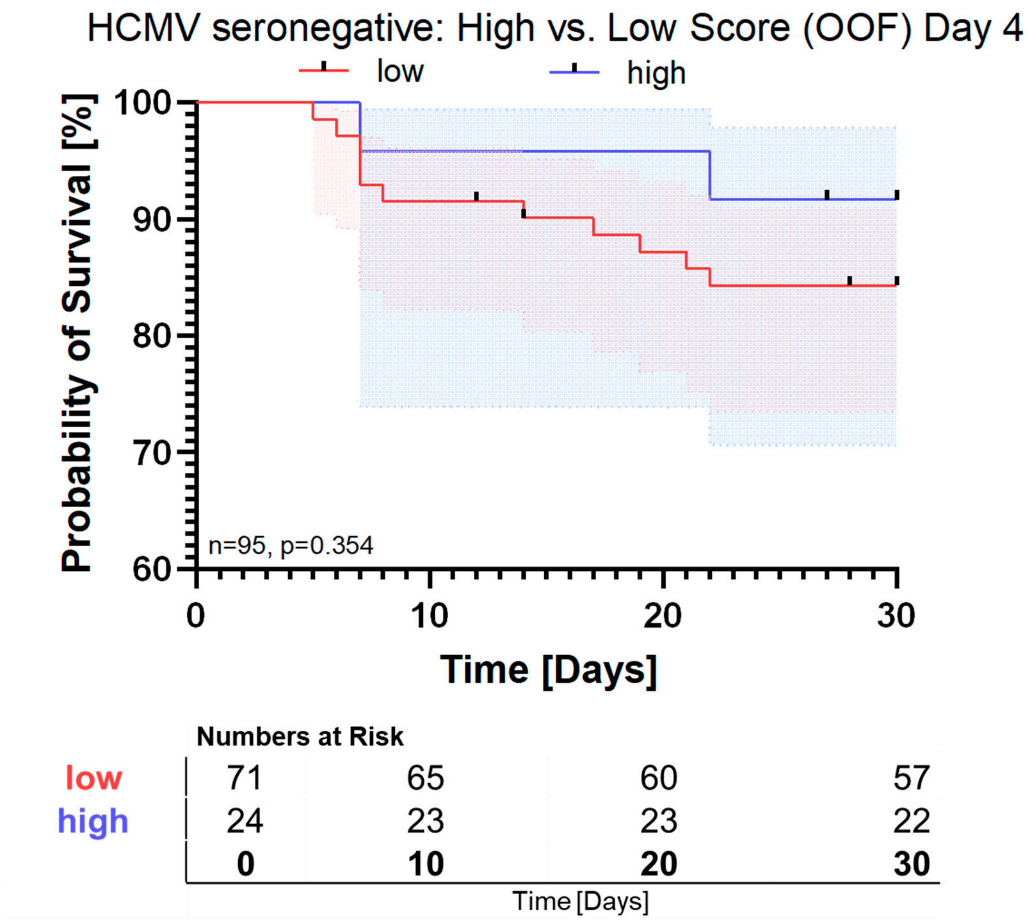

Supplementary Figure S5: Kaplan–Meier survival analysis of HCMV-seronegative patients based on model-predicted survival scores on Day 4. Kaplan–Meier curves depict 30-day survival probabilities for HCMV-seronegative patients, stratified into a low -score (red) and high -score (blue) group according to the logistic-regression model trained on 13 cytokines. Survival probabilities are derived from out-of-fold (OOF) predictions obtained during five-fold cross-validation. Shaded areas represent 95% confidence intervals. No significant difference was observed between groups (log-rank p = 0.354).

Supplementary Table S4: Baseline characteristic for patients with "High" Score and "Low" Score for the total cohort.

	**Total cohort**	**"High" Score**	**"Low" Score**	***p*-value**
n	**331 (100%)**	**143 (43.2%)**	**188 (56.8%)**	
Female sex, n (%)	118 (35.65%)	50 (35.7%)	68 (37.6 %)	0.822
Age, years (IQR)	66 (55 -76)	66 (55– 76)	66 (56–76)	0.866
SOFA at admission (IQR)	8 (5-11)	10 (6-13)	7 (5-10)	**<0.001**
ICU length of stay, days (IQR)	6.81 (2.53-14.45)	4.66 (1.87-11.90)	7.74 (3.07-15.00)	**0.008**
30 day survival, n (%)	237 (71.60%)	74 (39.86%)	42 (22.34%)	**<0.001**
**Laboratory values, day 1**				
C-reactive protein, mg/dL (IQR)	16.43 (9.56-26.69)	18.06 (9.77-26.59)	16.14 (9.47-26.70)	0.866
Procalcitonin, ng/mL (IQR)	2.54 (0.49-12.15)	7.02 (1.63-11.91)	1.02 (0.3-9.62)	**<0.001**
Lactate, mmol/L (IQR)	1.38 (0.96-2.04)	1.88 (1.34-4.05)	1.15 (0.89-1.60)	**<0.001**
Leukocytes, x10^3/µL (IQR)	13.4 (9.43-18.98)	13.1 (9.38-21.70)	13.85 (9.48-18.23)	0.598
**Comorbid condition, n (%)**	**272**			
Hypertension	179 (65.81%)	70 (63.06%)	109 (67.70%)	0.508
Cardiovascular Disease	108 (39.71%)	41 (36.94%)	67 (41.61%)	0.516
Chronic obstructive pulmonary disease	34 (12.50%)	15 (13.51%)	19 (11.80%)	0.822
Other lung disease	26 (9.56%)	13 (11.71%)	13 (8.07%)	0.428
Chronic kidney disease	52 (19.12%)	24 (21.62%)	28 (17.39%)	0.474
Malignancies	70 (25.74%)	29 (26.12%)	41 (25.47%)	0.999
Diabetes mellitus	78 (28.68%)	37 (33.33%)	41 (25.47%)	0.203
Obesity	78 (28.68%)	30 (27.03%)	48 (29.81%)	0.717
Dialysis	11 (4.04%)	5 (4.50%)	6 (3.73%)	0.994
Organ transplantation	31 (11.40%)	12 (10.81%)	19 (11.80%)	0.816
Nicotine abuse	52 (19.12%)	19 (17.11%)	33 (20.50%)	0.589
Alcohol abuse	22 (8.09%)	11 (9.91%)	11 (6.83%)	0.491
**Focus of infection, n (%)**	**299**	**129**	**170**	
Central nervous system	6 (2.00%)	3 (2.33%)	3 (1.76%)	0.999
Lower respiratory tract	135 (45.15%)	52 (40.31%)	83 (48.82%)	0.178
Skin and soft tissue	14 (4.68%)	6 (4.65%)	8 (4.71%)	0.999
Urinary tract	20 (6.69%)	7 (5.42%)	13 (7.65%)	0.598
Cardiovasculary system	16 (5.35%)	5 (3.88%)	13 (7.65%)	0.266
Intra-abdominal	75 (25.25%)	42 (32.56%)	33 (19.14%)	**0.014**
Musculoskeletal	11 (3.67%)	7 (5.42%)	4 (2.35%)	0.217
Other	18 (6.02%)	5 (3.88%)	13 (7.65%)	0.266
COVID-19	4 (1.33%)	2 (1.55%)	2 (1.18%)	0.999

Baseline demographic, clinical, and laboratory characteristics for patients with "High" Score and "Low" Score for the entire cohort (n = 331). Data are presented as median and interquartile range (IQR; q25-q75)) for continuous variables and as absolute and relative frequencies for categorical variables. HCMV serostatus was determined by ELISA on day 1 after study inclusion. P-values were calculated using the two-sided Mann–Whitney U test followed by false discovery rate (FDR) correction for continuous variables. Analysis of binary variables was performed using Chi2-test. Abbreviations: ICU = intensive care unit, SOFA = Sequential organ failure assessment score

Supplementary Table S5: Baseline characteristic for patients with “High” Score and “Low” Score for the seropositive group.

	**Total cohort**	**“High” Score**	**“Low” Score**	***p*-value**
n	**216**			
Female sex, n (%)	86 (40.72%)	32 (41.03%)	43 (43.88%)	0.821
Age, years (IQR)	67.00 (58.25-78)	67 (57.5–77)	67 (58.75–78)	0.837
SOFA at admission (IQR)	8 (6-11.25)	10.5 (7-14)	7 (5-10)	**<0.001**
ICU length of stay, days (IQR)	5.75 (2.18-14.15)	3.25 (1.87-11.91)	7.15 (3.07-15)	**0.014**
30 day survival, n (%)	143 (66.20%)	52 (53.06%)	88 (74.58%)	0.002
**Laboratory values, day 1**				
C-reactive protein, mg/dL (IQR)	18.4 (9.78-28.73)	19.77 (9.77–30.18)	16.32 (10.00–28.29)	0.915
Procalcitonin, ng/mL (IQR)	2.93 (0.59-12.21)	7.75 (1.93–17.52)	1.33 (0.34–10.40)	**<0.001**
Lactate, mmol/L (IQR)	1.53 (1.00-2.44)	1.99 (1.40–6.08)	1.19 (0.88–1.69)	**<0.001**
Leukocytes, x10^3/µL (IQR)	13.05 (8.93-19.18)	13.45 (9.31–20.99)	12.75 (8.69–17.73)	0.278
**Comorbid condition, n (%)**	**176**			
Hypertension	117 (66.48%)	10 (12.82%)	16 (16.33%)	0.662
Cardiovascular Disease	75 (42.61%)	27 (34.62%)	30 (30.61%)	0.688
Chronic obstructive pulmonary disease	20 (11.36%)	10 (12.82%)	10 (10.20%)	0.761
Other lung disease	14 (7.95%)	49 (62.82%)	68 (69.39%)	0.450
Chronic kidney disease	38 (21.59%)	19 (24.36%)	19 (19.39%)	0.541
Malignancies	47 (26.70%)	5 (6.41%)	8 (8.16%)	0.879
Diabetes mellitus	57 (32.39%)	24 (30.77%)	23 (23.47%)	0.360
Obesity	52 (29.55%)	12 (15.38%)	18 (18.37%)	0.748
Dialysis	7 (3.98%)	7 (8.97%)	7 (7.14%)	0.868
Organ transplantation	26 (14.77%)	22 (28.21%)	30 (30.61%)	0.856
Nicotine abuse	30 (17.05%)	4 (5.13%)	3 (3.06%)	0.701
Alcohol abuse	13 (7.39%)	37 (38.95%)	49 (43.75%)	0.577
**Focus of infection, n (%)**	**193**			
Central nervous system	3 (1.55%)	2 (2.30%)	1 (0.94%)	0.590
Lower respiratory tract	84 (43.52%)	36 (41.38%)	48 (45.28%)	0.690
Skin and soft tissue	12 (6.21%)	6 (6.90%)	6 (5.66%)	0.957
Urinary tract	12 (6.21%)	4 (4.60%)	8 (7.55%)	0.552
Cardiovascular system	12 (6.21%)	5 (5.75%)	7 (6.60%)	1.000
Intra-abdominal	48 (24.87%)	25 (28.74%)	23 (21.70%)	0.338
Musculoskeletal	5 (2.59%)	3 (3.45%)	2 (1.89%)	0.659
Other	13 (6.74%)	4 (4.60%)	9 (8.49%)	0.390
COVID-19	4 (2.07%)	2 (2.30%)	2 (1.89%)	1.000

Baseline demographic, clinical, and laboratory characteristics for patients with “High” Score and “Low” Score for the seropositive subgroup (n = 216). Data are presented as median and interquartile range (IQR; q25-q75)) for continuous variables and as absolute and relative frequencies for categorical variables. HCMV serostatus was determined by ELISA on day 1 after study inclusion. P-values were calculated using the two-sided Mann–Whitney U test followed by false discovery rate (FDR) correction for continuous variables. Analysis of binary variables was

performed using Chi2-test. Abbreviations: ICU = intensive care unit, SOFA = Sequential organ failure assessment score.

Supplementary Table S6: Baseline characteristic for patients with "High" Score and "Low" Score for the seronegative group.

	Total cohort	"High" Score	"Low" Score	*p*-value
n	**115**			
Female sex, n (%)	32 (29.06%)	13 (28.89%)	19 (27.54%)	0.999
Age, years (IQR)	62.5 (54-73)	61 (54–74)	63 (54–73)	0.765
SOFA at admission (IQR)	7.5 (5-10.75)	9 (4-12)	7 (5-10)	0.617
ICU length of stay, days (IQR)	7.33 (3.01-14.6)	6.54 (2.40-11.90)	7.88 (3.45-14.90)	0.350
30 day survival, n (%)	94 (81.73%)	34 (75.56%)	58 (82.86%)	0.474
**Laboratory values, day 1**				
C-reactive protein, mg/dL (IQR)	14.88 (8.69-24.41)	14.88 (8.69–24.41)	14.97 (8.69–22.03)	0.632
Procalcitonin, ng/mL (IQR)	1.66 (0.27-11.16)	5.6 (0.97–19.72)	0.72 (0.20–6.11)	0.029
Lactate, mmol/L (IQR)	1.21 (0.93-1.83)	1.84 (1.21–2.40)	1.10 (0.90–1.29)	**<0.001**
Leukocytes, x10^3/µL (IQR)	15.1 (9.78-18.8)	12.2 (9.5–22.4)	16.5 (10.69–18.31)	0.584
**Comorbid condition, n (%)**	**96**			
Hypertension	62 (64.58%)	21 (63.64%)	41 (65.08%)	0.999
Cardiovascular Disease	33 (34.38%)	9 (27.27%)	24 (38.10%)	0.404
Chronic obstructive pulmonary disease	14 (14.58%)	5 (15.15%)	9 (14.29%)	0.999
Other lung disease	12 (12.50%)	6 (18.18%)	6 (9.52%)	0.372
Chronic kidney disease	14 (14.58%)	5 (15.15%)	9 (14.29%)	0.999
Malignancies	23 (23.96%)	5 (15.15%)	18 (28.57%)	0.226
Diabetes mellitus	21 (21.88%)	10 (30.30%)	11 (17.46%)	0.236
Obesity	26 (27.08%)	8 (24.24%)	18 (28.57%)	0.832
Dialysis	4 (4.17%)	1 (3.03%)	3 (4.76%)	0.999
Organ transplantation	5 (5.21%)	2 (6.06%)	3 (4.76%)	0.999
Nicotine abuse	22 (22.92%)	7 (21.21%)	15 (23.81%)	0.975
Alcohol abuse	9 (9.38%)	6 (18.18%)	3 (4.76%)	0.059
**Focus of infection, n (%)**	**106**			
Central nervous system	3 (2.83%)	1 (2.38%)	2 (3.13%)	0.999
Lower respiratory tract	51 (48.11%)	16 (38.10%)	35 (54.69%)	0.141
Skin and soft tissue	2 (1.88%)	0 (0%)	2 (3.13%)	0.517
Urinary tract	8 (7.55%)	3 (7.14%)	5 (7.81%)	0.999
Cardiovascular system	4 (3.77%)	0 (0%)	4 (6.25%)	0.150
Intra-abdominal	27 (25.47%)	17 (40.48%)	10 (15.63%)	0.008
Musculoskeletal	6 (5.66%)	4 (9.52%)	2 (3.13%)	0.211
Other	5 (4.71%)	1 (2.38%)	4 (6.25%)	0.646
COVID-19	0 (0 %)	0 (0%)	0 (0%)	0.999

Baseline demographic, clinical, and laboratory characteristics for patients with "High" Score and "Low" Score for the seronegative subgroup (*n* = 115). Data are presented as median and interquartile range (IQR; q25-q75)) for continuous variables and as absolute and relative frequencies for categorical variables. HCMV serostatus was determined by ELISA on day 1 after study inclusion. P-values were calculated using the two-sided Mann–Whitney U test followed by false discovery rate (FDR) correction for continuous variables. Analysis of binary variables was

performed using Chi2-test. Abbreviations: ICU = intensive care unit, SOFA = Sequential organ failure assessment score.
